# In the national epidemiological bulletins – a selection from recent issues

**DOI:** 10.2807/1560-7917.ES.2017.22.39.170928-1

**Published:** 2017-09-28

**Authors:** Eurosurveillance editorial team

**Affiliations:** 1 European Centre for Disease Prevention and Control (ECDC), Stockholm, Sweden

**Keywords:** infectious diseases, public health, Europe

**Healthcare-associated infections and antibiotic resistance**

Laboratory surveillance of candidaemia in England, Wales and Northern Ireland: 2016

Health Protection Report; 11(32)

15 September 2017, United Kingdom

https://www.gov.uk/government/uploads/system/uploads/attachment_data/file/645312/hpr3217_cnddmia2016.pdf

Laboratory surveillance of *Staphylococcus aureus* bacteraemia in England, Wales and Northern Ireland: 2016

Health Protection Report; 11(29)

18 August 2017, United Kingdom

https://www.gov.uk/government/uploads/system/uploads/attachment_data/file/638891/hpr2917_stph-aurs.pdf

Laboratory surveillance of *Pseudomonas* spp. and *Stenotrophomonas* spp. bacteraemia in England, Wales and Northern Ireland: 2016

Health Protection Report; 11(25)

21 July 2017, United Kingdom

https://www.gov.uk/government/uploads/system/uploads/attachment_data/file/631374/hpr2517_psdmns.pdf

**Food- and waterborne diseases**

Epidemiological and microbiological investigation of temporally parallel food-related *Salmonella* Kottbus events

Epidemiologisches Bulletin 38, 2017

21 September 2017, Germany

https://www.rki.de/DE/Content/Infekt/EpidBull/Archiv/2017/Ausgaben/38_17.pdf?__blob=publicationFile

Hepatitis A outbreak linked to the contamination of products from a bakery, Herault (France), 2014

Bulletin épidémiologique hebdomadaire, 21

19 September 2017, France

http://invs.santepubliquefrance.fr/beh/2017/21/pdf/2017_21.pdf

Incidence of protozoal infection reported to HPS in 2016

HPS Weekly Report 2017; 51(37)

19 September 2017, Scotland

http://www.hps.scot.nhs.uk/documents/ewr/pdf2017/1737.pdf

Trends in *Salmonella* in the Netherlands in 2016. In humans, farm animals and in food

Infectieziekten Bulletin 2017; 28(7)

14 September 2017, the Netherlands

http://www.rivm.nl/Documenten_en_publicaties/Algemeen_Actueel/Uitgaven/Infectieziekten_Bulletin/Jaargang_28_2017/Infectieziekten_Bulletin_jaargang_28_nummer_7_september_2017/Inhoud_september_2017/Trends_in_Salmonella_in_Nederland_in_2016_Bij_de_mens_landbouwhuisdieren_en_in_voedsel

Surveillance of shigatoxin-producing *Escherichia coli *(STEC) in the Netherlands, 2016

Infectieziekten Bulletin 2017; 28(7)

14 September 2017, the Netherlands

http://www.rivm.nl/Documenten_en_publicaties/Algemeen_Actueel/Uitgaven/Infectieziekten_Bulletin/Jaargang_28_2017/Infectieziekten_Bulletin_jaargang_28_nummer_7_september_2017/Inhoud_september_2017/Surveillance_van_shigatoxine_producerende_Escherichia_coli_STEC_in_Nederland_2016

STEC in Scotland 2016: enhanced surveillance and reference laboratory data

HPS Weekly Report 2017; 51(32)

15 August 2017, Scotland

http://www.hps.scot.nhs.uk/documents/ewr/pdf2017/1732.pdf

**Hepatitides**

Acute hepatitis B (England): annual report for 2016

Health Protection Report; 11(31)

8 September 2017, United Kingdom

https://www.gov.uk/government/uploads/system/uploads/attachment_data/file/643558/hpr3117_hepB_ann.pdf

Laboratory reports of hepatitis A and C in England and Wales, January to March 2017

Health Protection Report; 11(31)

8 September 2017, United Kingdom

https://www.gov.uk/government/uploads/system/uploads/attachment_data/file/643539/hpr3117_hepAC.pdf

Viral hepatitis B and D in the year 2016

Epidemiologisches Bulletin 31, 2017

3 August 2017, Germany

https://www.rki.de/DE/Content/Infekt/EpidBull/Archiv/2017/Ausgaben/31_17.pdf?__blob=publicationFile

Hepatitis C in the year 2016

Epidemiologisches Bulletin 30, 2017

27 July 2017, Germany

https://www.rki.de/DE/Content/Infekt/EpidBull/Archiv/2017/Ausgaben/30_17.pdf?__blob=publicationFile

**Vaccine-preventable diseases**

Vaccination coverage, knowledge, perceptions and attitudes of teenagers toward vaccination in Calvados and Orne districts (Normandy, France), 2015–2016

Bulletin épidémiologique hebdomadaire, 21

19 September 2017, France

http://invs.santepubliquefrance.fr/beh/2017/21/pdf/2017_21.pdf

Epidemiology of invasive group B streptococcal infection in infants in Ireland: 2012–2017

Epi-Insight 2017;18(9)

September 2017, Ireland

http://ndsc.newsweaver.ie/epiinsight/nk1rq5laued1qsl0rhv973?a=2&p=52270887&t=17517804

Invasive pneumococcal disease and the impact of pneumococcal conjugate vaccines in Ireland, 2008-2016

Epi-Insight 2017;18(8)

August 2017, Ireland

http://ndsc.newsweaver.ie/epiinsight/10namozldoj?a=2&p=52157231&t=17517804

Laboratory confirmed cases of measles, mumps and rubella, England: April to June 2017

Health Protection Report; 11(30)

25 August 2017, United Kingdom

https://www.gov.uk/government/uploads/system/uploads/attachment_data/file/640460/hpr3017_mmr.pdf

Increase of invasive infections with group A streptococci in January–April 2017

EPI-NEWS 27a+b, 2017

5 July 2017, Denmark

https://www.ssi.dk/Aktuelt/Nyhedsbreve/EPI-NYT/2017/Uge%2027a%20-%202017.aspx

Invasive meningococcal disease (laboratory reports in England): January to March 2017

Health Protection Report; 11(23)

30 June 2017, United Kingdom

https://www.gov.uk/government/uploads/system/uploads/attachment_data/file/624164/hpr2317_IMD.pdf

Laboratory confirmed cases of Pertussis (England): January to March 2017Health Protection Report; 11(23)

30 June 2017, United Kingdom

https://www.gov.uk/government/uploads/system/uploads/attachment_data/file/624162/hpr2317_prtsss.pdf

Quarterly vaccination coverage statistics for children aged up to five years in the UK (COVER programme): January to March 2017Health Protection Report; 11(23)

30 June 2017, United Kingdom

https://www.gov.uk/government/uploads/system/uploads/attachment_data/file/623860/hpr2317_COVER.pdf

Pneumococcal Polysaccharide Vaccine (PPV) coverage report, England, April 2016 to March 2017

Health Protection Report; 11(23)

30 June 2017, United Kingdom

https://www.gov.uk/government/uploads/system/uploads/attachment_data/file/624133/hpr2317_PPV.pdf

**Respiratory diseases**

Legionellosis in Scotland 2015–2016

HPS Weekly Report 2017; 51(34)

29 August 2017, Scotland

http://www.hps.scot.nhs.uk/documents/ewr/pdf2017/1734.pdf

**Sexually transmitted diseases**

HIV infection and AIDS: Quarterly report to 30 June 2017

HPS Weekly Report 2017; 51(36)

12 September 2017, Scotland

http://www.hps.scot.nhs.uk/documents/ewr/pdf2017/1736.pdf

HIV 2016

EPI-NEWS 36, 2017

6 September 2017, Denmark

https://www.ssi.dk/Aktuelt/Nyhedsbreve/EPI-NYT/2017/Uge%2036%20-%202017.aspx

Screening of pregnant women for hepatitis B, HIV and syphilis, 2016

EPI-NEWS 35, 2017

30 August 2017, Denmark

https://www.ssi.dk/Aktuelt/Nyhedsbreve/EPI-NYT/2017/Uge%2035%20-%202017.aspx

Chlamydia 2016

EPI-NEWS 34, 2017

23 August 2017, Denmark

https://www.ssi.dk/Aktuelt/Nyhedsbreve/EPI-NYT/2017/Uge%2034%20-%202017.aspx

Syphilis in Scotland 2016: update

HPS Weekly Report 2017; 51(33)

22 August 2017, Scotland

http://www.hps.scot.nhs.uk/documents/ewr/pdf2017/1733.pdf

Sexually transmitted infections: updating data of two sentinel surveillance systems active in Italy on 31 December 2015

Not Ist Super Sanità 2017;30(7-8)

July−August 2017, Italy

http://www.iss.it/binary/publ/cont/ONLINE_lug_ago_2017.pdf

Sentinel surveillance of sexually transmitted infections based on clinical microbiology laboratories

Not Ist Super Sanità 2017;30(7-8)

July−August 2017, Italy

http://www.iss.it/binary/publ/cont/ONLINE_lug_ago_2017.pdf

Annual report from the sentinel surveillance study of blood borne virus testing in England: data for January to December 2016

Health Protection Report; 11(26)

28 July 2017, United Kingdom

https://www.gov.uk/government/uploads/system/uploads/attachment_data/file/633797/hpr2617_bbv-ss.pdf

HIV prevalence estimate among men who have sex with men attending gay venues in five French cities – PREVAGAY 2015

Bulletin épidémiologique hebdomadaire, 18

18 July 2017, France

http://invs.santepubliquefrance.fr/beh/2017/18/pdf/2017_18.pdf

Outbreak of hepatitis A among men who have sex with men, Rouen (France), December 2016 - April 2017

Bulletin épidémiologique hebdomadaire, 18

18 July 2017, France

http://invs.santepubliquefrance.fr/beh/2017/18/pdf/2017_18.pdf

**Zoonoses and vector-borne diseases**

Common animal associated infections quarterly report (England and Wales): second quarter 2017

Health Protection Report; 11(28)

11 August 2017, United Kingdom

https://www.gov.uk/government/uploads/system/uploads/attachment_data/file/637401/hpr2817_zoos.pdf

Travel health: Malaria reported in Scotland 2016

HPS Weekly Report 2017; 51(28)

18 July 2017, Scotland

http://www.hps.scot.nhs.uk/documents/ewr/pdf2017/1728.pdf

**Other**

Infectious disease surveillance of migrant populations in Calais and Grande-Synthe, France, November 2015 – October 2016

Bulletin épidémiologique hebdomadaire, 19-20

5 September 2017, France

http://invs.santepubliquefrance.fr/beh/2017/19-20/pdf/2017_19-20.pdf

Creutzfeldt-Jakob disease (CJD) biannual update (August 2017)

Health Protection Report; 11(29)

18 August 2017, United Kingdom

https://www.gov.uk/government/uploads/system/uploads/attachment_data/file/640136/hpr2917_CJD.pdf

Seroprevalence of HIV infections, syphilis, hepatitis B, hepatitis C and hepatitis A in asylum seekers in Saxony

Epidemiologisches Bulletin 29, 2017

20 July 2017, Germany

https://www.rki.de/DE/Content/Infekt/EpidBull/Archiv/2017/Ausgaben/29_17.pdf?__blob=publicationFile

